# Uterine rupture among mothers admitted for obstetrics care and associated factors in referral hospitals of Amhara regional state, institution-based cross-sectional study, Northern Ethiopia, 2013-2017

**DOI:** 10.1371/journal.pone.0208470

**Published:** 2018-12-04

**Authors:** Worku Taye Getahun, Abayneh Aklilu Solomon, Fisseha Yetewale Kassie, Habtamu Kebebe Kasaye, Habtamu Temesgen Denekew

**Affiliations:** 1 Department of Midwifery, Debremarkos Referral Hospital, Debremarkos, Ethiopia; 2 School of Midwifery, University of Gondar, Gondar, Ethiopia; 3 Department of Midwifery, Institute of Health Sciences, Wollega University, Nekemte, Ethiopia; 4 Departments of Human Nutrition, Debremarkos University, Debremarkos, Ethiopia; The University of Warwick, UNITED KINGDOM

## Abstract

**Background:**

Maternal morbidity and mortality have been one of the most challenging health problems that concern the globe over the years. Uterine rupture is one of the peripartum complications, which cause nearly about one out of thirteen maternal deaths. This study aimed to assess the prevalence and associated factors of uterine rupture among obstetric case in referral hospitals of Amhara Regional State, Northern Ethiopia.

**Methods:**

Institution based cross sectional study was conducted from Dec 5-2017-Jan 5–2018 on uterine rupture. During the study randomly selected 750 charts were included by using simple random sampling method. Data were checked, coded and entered into Epi info version 7.2 and then exported to SPSS Version 20 for Analysis. Binary Logistic regression was used to identify the predictors of uterine rupture and 95% Confidence Interval of odds ratio at p-value less than 0.05 was taken as a significance level.

**Result:**

The overall prevalence of uterine rupture was 16.68% (95% CI: 14%, 19.2%). Distance from health facility >10km (Adjusted Odds Ratio (AOR) = 2.44; 95%CI:1.13,5.28), parity between II and IV (AOR = 7.26;95% (3.06,17.22)) and ≥V (AOR = 12.55;95% CI 3.64,43.20), laboring for >24hours(AO = 3.44; 95% CI:1.49,7.92), with referral paper(AOR = 2.94;95%CI:1.28,6.55) diagnosed with obstructed labor (AOR = 4.88;95%CI: 2.22,10.70), precipitated labor (AOR = 3.59;95%CI:1.10,11.77), destructive delivery (AOR = 5.18;95%: 1.22,20.08), No partograph (AOR = 5.21; 95% CI: 2.72,9.97), CPD(AOR = 4.08;95%CI:1.99,8.33), morbidly adherent placenta (AOR = 9.00;95%:2.46,27.11), gestational diabetic militias (AOR = 5.78; 95%CI:1. 12,20 .00 ), history of myomectomy(AOR = 5.00;95%CI:1.33,18.73), induction and augmentation of labor (AOR = 2.34;95%:1.15,4.72) obstetric procedure (AOR = 2.54;95%: 1.09,5.91), previous caesarian deliveries 4.90 (2.13,11.26) were found to be significantly associated with uterine rupture.

**Conclusion:**

This finding showed that the prevalence of uterine rupture is higher. A more vigilant approach to prevent prolonged and obstructed labor, use of partograph, quick referral to a well-equipped center and prevention of other obstetrics complications need to be focused on.

## Introduction

Uterine rupture is a ripping of the uterine wall during pregnancy, delivery or immediately after childbirth. Commonly occurs in the lower segment of the uterus during the intrapartum or antepartum period from any causes[1]. Many women shoulder a dual liability of supporting the whole family by working outside the home and taking full responsibility for household’s duties and childcare. Although women in the community play vital roles, the health requirements of women are neglected as it could be evidenced by the soaring level of maternal mortality in many developing countries[2].

Worldwide, nearly 340,000 to half a million women die per annum due to complications of pregnancy and childbirth. Maternal mortality and morbidity remain extremely high health problem and challenge that concerns the globe over the years across developing countries especially in sub-Saharan African (SSA) and South Asia. About 99% of maternal death is occurring in developing countries due to preventable causes. More than 87% of maternal deaths from the global Maternal Mortality ratio, which is estimated to be 210 deaths per 100, 000 live births in 2013, are accounted by sub-Saharan Africa and South Asia[3].

Ethiopia is one of the countries with the highest maternal mortality (MM), which is located in sub-Saharan Africa where more than half of the global MM ensue [4,5]. This high maternal mortality occurs due to different direct and indirect causes that indicate poor quality maternity continuum of care.

Globally, the incidence of uterine rupture is 0.07%, which is much lower than the magnitude encountering in Africa-1.3%[6]. Uterine rupture is one of the peripartum complications that causes nearly about one out of thirteen maternal death and the remaining survivors encounter immediate and long-term complexities[7]. In Ethiopia, uterine rupture and obstructed labor together account for 29% of the total maternal mortality. These are the significant obstetric sequel needing concern, as they contributed the second significant causes of maternal death next to abortion-related complications[8].

There are contradictions among works of literature regarding factors that lead to uterine rupture. Factors that determine uterine rupture varies across setting because maternal characteristics also change, so do the health services delivery system and reception [9].

Uterine rupture would not only have limited to just short-term complications, and it ends up with long-term complications, maternal mortality and perinatal mortality. Even though uterine rupture contributes to the maternal mortality, there is the limitation of substantial evidence indicating the level and factors contributing to uterine rupture for prevention and life-saving measure in health facilities. To enable this, we tried to assess the prevalence of uterine rupture and its predictors in the study area.

## Methodology

### Study setting and population

An Institution based cross-sectional study was conducted in referral hospitals of Amhara regional state from, December 5, 2017, to January 5, 2018. Amhara regional state is found between 11° 30' 00" N latitude & 38° 30' 00" E longitude on the northwestern part of Ethiopia. This region has a total catchment area population of 17,221,976 of whom 8,641,580 were men and 8,580,396 women and from which urban inhabitants account 2,112,595(12.27%) of the population[10].

Sixty-seven hospitals, 839 health centers and 3336 health posts found in Amhara regional state. Among these five of the hospitals are referral hospitals, which include Debrebirhan Referral Hospital, Felege-Hiwot referral hospital, Debre-Markos Referral Hospital, Dessie Referral Hospital and University of Gondar Teaching and Referral Hospital. The data used for the analysis of this study were abstracted from the charts (medical records) of mothers who had taken obstetrics care within the third trimester of their pregnancy at Referral Hospitals of Amhara regional state from 2013 to 2017.

### Sample size and sampling procedure

A total of 756 sample size was calculated by using Epi Info Stat Calc version 7.2 population survey by taking assumptions of population size >10,000, 95% confidence level, prevalence of uterine rupture (9.5%) from previous study[1], margin of error 3%, design effect two and 2.9% non-response rate. A multistage sampling technique was employed to select the hospitals & study participants with the assumptions of homogeneity of service in Amhara regional state referral hospitals. Felege Hiwot referral hospital, Debre Markos Referral Hospital and Dessie Referral hospital were selected. The charts were allocated to the proportion of the patient flow in each hospital and the charts were chosen by simple random sampling technique using the table of random number based on medical record numbers presenting within study duration.

### Data collection procedures and instrumentation

English version of data abstraction checklist, which we adapted from various literatures, was used to collect data from clients’ medical records and it has two parts, the first part deals with sociodemographic characteristics whereas the second part contains obstetric characteristics. Six BSc midwives collected data and three additional senior BSc midwives supervised the data collection process. Data collection were done by these midwives who were from health centers in order to minimize social desirability bias, two data collectors and one supervisor for each hospital were assigned.

### Data quality control

To assure the data quality high emphasis was given in designing data abstraction tool. The pretest was conducted on 5% of the sample size at Debre Markos hospital and necessary correction on the instrument was employed accordingly. One-day training was provided for data collectors and supervisors regarding the objectives of the study, data collection methods, the significance of the study, data collection tool, ethical considerations and way of abstraction of necessary information from the chart. During data collection, assigned supervisors visited and supervised the data collection process and checked the completeness of the extracted tool.

### Data processing and analysis

All collected questioners were rechecked for completeness and coded. Then the data were entered and cleaned using Epi Info 7.2 software, and exported to SPSS version 20 for further analysis. Descriptive statistics like frequencies and cross tabulation were carried out to characterize the study population using socio-demographic and obstetric variables.

Bivariable logistic regression was employed to identify the association between dependent and independent variables, those variables having a p-value of <0.05 in the bivariable analyses were fitted into multivariable logistic regression analysis with enter method to control the effects of confounding factors. Ninety-five percent confidence interval of odd ratios was computed and a p-value of less than 0.05 was considered to declare the statistical significance. The assumption of binary logistic regression model was checked by using Hosmer and Lemeshow test of goodness of fit test. Tables and graphical presentation were used to present the findings of the study.

### Ethical consideration

Ethical clearance letter was obtained from the Ethical Review Committee of the Department of Midwifery, under the delegation of the Institution Review Board of the University of Gondar with a reference number of MIDW/10/489/2010. The IRB waived the requirements of written informed consent as the data used were secondary data from a patients’ records. Up on bearing with ethical review, administration of each Referral Hospitals provided us permission to access the patients’ medical records. Privacy and confidentiality of all information were kept by coding throughout the research work.

## Result

### Socio-demographic characteristics

Out of 756 charts selected for review, we included 750 cases with complete information from three hospitals in the analysis. The ages of mothers who got uterine rupture ranged between 18–42 years, with a mean age of 27.78 years and SD 5.887. Most of the women 647(86.26%) were in the age range between 19 years to 35 years, while 715 (95.30%) were Amhara in ethnicity. About 521(69.5%) mothers lived >10 kilometers (km) from the health institutions. The average distance they traveled was 44.05 km with SD of 38.308 km. Most of the uterine rupture (113 (90.40%)) occurred from mothers those who lived in area presenting at above 10km from health facility they received care (Table 1).

**Table 1:** Socio-demographic characteristics among women with the obstetric case, in Amhara region referral hospitals, Ethiopia, 2017.

Variables	Categories	Uterine rupture		Frequency (n = 750)	(%)
		Yes	No		
Age in years	< = 18yrs	2	19	21	2.8
	19-35yrs	98	549	647	86.2
	> = 35yrs	25	57	82	11.00.
Ethnicity	Amhara	117	598	715	95.30
	Tigry	2	15	17	2.30
	Oromo	4	6	10	1.30
	Others*	2	6	8	1.10
Distance	< = 10km	12	217	229	30.5
	>10km	113	408	521	69.5

*Afar and Gumuz

### Obstetric characteristics

The parity ranges from one to eleven with the mean parity of 2.59 ± SD 1.59. Among the patients who the uterine rupture, the majority (81.6%) of the patients were of para two up to four. About 621(93.8%) mothers had ANC follow up two and above and forty-one (6.2%) had less than two ANC for indexed pregnancy. Most of the mothers came with referred papers 506 (67.5%), and 469(92.86%) were referred from a health institution while the remaining 37(7.3%) were self-referral. Regarding the distributions of the previous mode of delivery in the hospitals, 516 women were multiparous with 460 (89.14%) spontaneous vertex delivery, 31 (6%) cesarean delivery and 25 (4.86%) both SVD and Cesarean delivery. One hundred thirty-one (17.3%) women gave birth with Assisted Instrumental Delivery, with 101 (77.1%) vacuum and 30 (22.9%) forceps delivery.

Fifty-six (7.5%) women had a history of the previous cesarean section, among them, 41 (73.2%) discharged before the seventh postoperative day. Among 190 (25.33%) mothers who were induced /augmented, 36 (18.9%) were induced /augmented for less than two hours. Most of the women were in their term gestational age, 615(82%) with the mean gestational age of 39.51wks ±1.94 SD. Regarding the presentation of the fetus, 633 (84.4%) were vertex, and 542 (72.26%) women followed with Partograph during labor. There were about 13 (1.73%) destructive deliveries 11 (84.6%) craniotomy, one evisceration and one decapitation. About 131 (17.46%) women developed Cephalo Pelvic disproportion and 167 (22.3%) women had a traumatic vaginal birth. Fifteen mothers were had morbidly adherent placenta with most of them fail in the category of placenta accreta 7(46.7%) (Table 2).

Among mothers having ANC follow up, 96 (14.50%) were diagnosed with uterine rupture and from these 89 (92.7%) ruptured cases had ANC visit two and above. Among patients with uterine rupture, 84 (67.2%) of them have stayed in labor for less than 24 hours with a mean duration of labor 15.14hours and SD of 8.038. The majority of ruptured case 112(89.6%) came with referred papers.

From the women having the history of cesarean section 20 (16%) experienced the uterine rupture. Among mothers with a history of the previous cesarean section, 15 (26.8%) got pregnant within less than six-month interval from the last cesarean delivery time, from which more than half 11 (55%) end up with uterine rupture. Likewise inter delivery interval out of 56 previous cesarean deliveries 20 (35.7%) were delivered within less than 18 months of their last birth. From 56 mothers with previous cesarean deliveries, 20 had developed postoperative fever, of them 16 (80%) developed uterine rupture.

More than two out of three vertex presentation (68.8%) end up with uterine rupture, and most of them were while their gestational age was term 100(80%). Out of ruptured cases, 82 (65.6%) laboring women not followed with partograph throughout labor. From women exposed to obstructed labor, almost half of them (47.2%) experienced the uterine rupture. Sixty-eight (54.4%) women those experienced CPD and 39(31.2%) victim of traumatic childbirth got uterine rupture (Table 2, in the third column).

**Table 2:** Obstetric characteristics with the distribution of uterine rupture among women with the obstetric case in Amhara region referral hospitals, Ethiopia, 2017.

Variables	Categories	Uterine rupture(%) (n = 125)	Total frequency(%) (n = 750)
**Parity**	Primipara	8(6.4)	235(31.3)
	Two-four	102(81.6)	476(63.5)
	Above four	15(12)	39(5.2)
**Aantenatal Care**	Yes	96(76.8)	662(88.3)
	No	29(23.2)	88(11.7)
**Duration of labor**	<24hrs	84(67.2)	661(88.1)
	≥24hrs	41(32.8)	89(11.9)
**Referral paper**	Yes	112(89.6)	506(67.5)
	No	13(10.4)	244(32.5)
**Partograph used**	Yes	43(34.4)	542(72.3)
	No	82(65.6)	208(27.7)
**Fetal presentation**	Vertex	84(67.2)	633(84.4)
	breech	11(8.8)	30(4.0)
	Face	16(12.8)	32(4.3)
	Brow	4(3.2)	39(5.2)
	Compound or shoulder	10(8.0)	16(2.1)
**Obstructed labor**	Yes	59(47.2)	92(12.3)
	No	66(52.8)	658(87.7)
**Gestational Age**	<37wks	12(9.6)	104(13.9)
	37-42wks	100(80.0)	615(82.0)
	≥42wks	13(10.4)	31(4.1)
**Induction /augmentation**	Yes	45(36.0)	190(25.3)
	No	80(64.0)	560(74.7)
**Birth weight**	<4000gm	110(88.0)	712(94.9)
	≥4000gm	15(12.0)	38(5.1)
**Destructive delivery**	Yes	5(4.0)	13(1.7)
	No	120(96.0)	737(98.3)
**Traumatic vaginal birth**	Yes	39(31.2)	167(22.3)
	No	86(68.8)	583(77.7)
**Cephalo Pelvic Disproportions**	Yes	68(54.4)	131(17.5)
	No	57(45.6)	619(82.5)
**Obstetric procedure**	Yes	13(10.4)	54(7.2)
	No	112(89.6)	696(92.8)
**Adherent placenta**	Yes	6(4.8)	15(2.0)
	No	119(95.2)	735(98.0)
**Precipitated labor**	Yes	15(12.0)	37(4.9)
	No	110(88.0)	713(95.1)
**Gestational Diabetes Mellitus**	Yes	16(12.8)	35(4.7)
	No	109(87.2)	715(95.3)
**Multiple pregnancy**	Yes	15(12.0)	38(5.1)
	No	110(88.0)	712(94.9)
**Previous myomectomy**	Yes	6(4.8)	16(2.1)
	No	119(95.2)	734(97.9)
**Previous caesarean delivery**	Yes	20(16.0)	56(7.5)
	No	105(84.0)	694(92.5)
**Instrumental delivery**	Yes	34(27.2)	131(17.5)
	No	91(72.8)	619(82.5)

### Prevalence of uterine rupture

**Fig 1:**
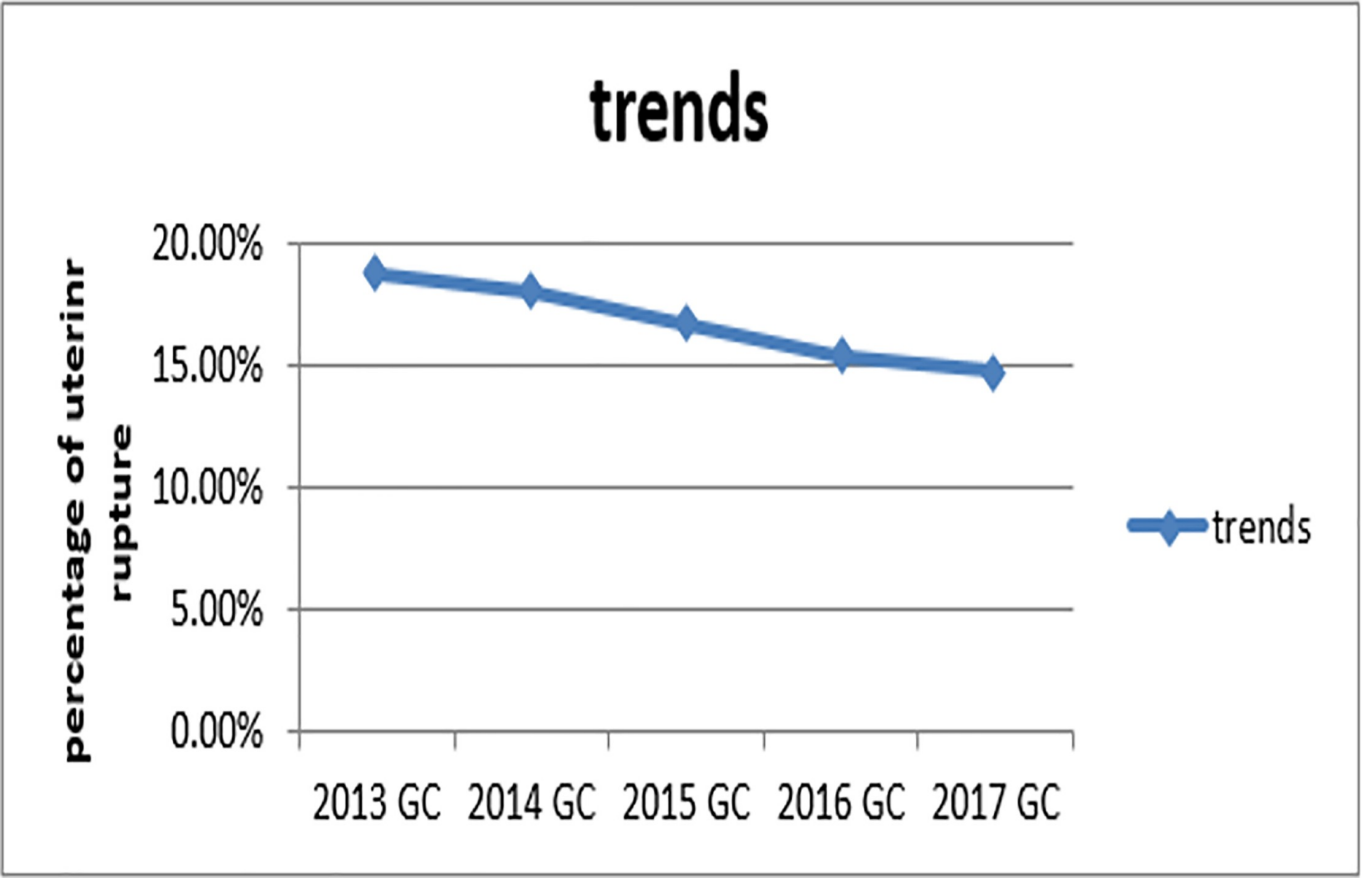
Trends of uterine rupture from 2013–2017 among mothers who got obstetric care in Amhara region referral hospitals, Northern Ethiopia, 2017.

**Fig 2:**
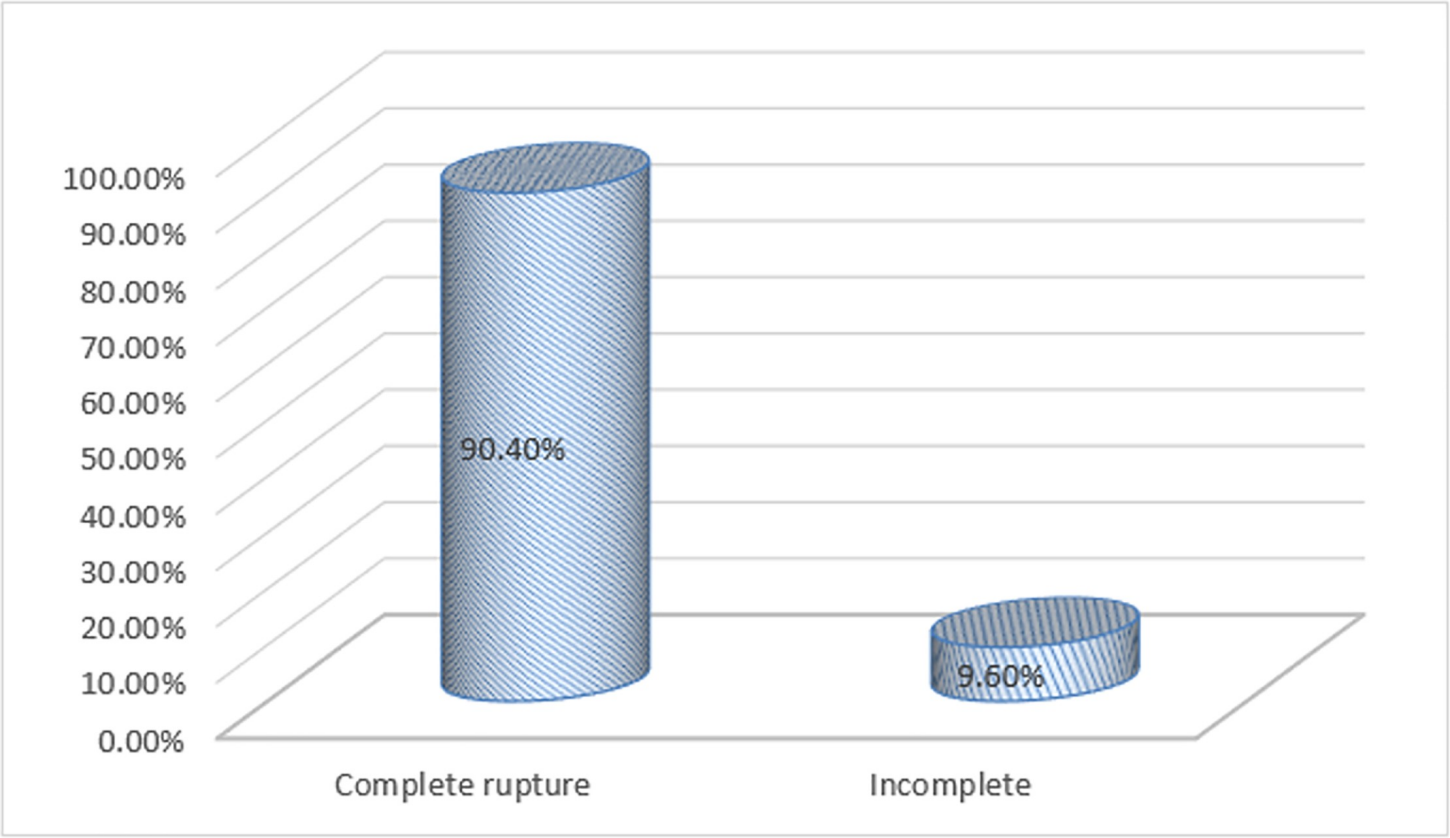
Types of uterine rupture among mothers at Amhara region referral hospitals Northern Ethiopia, 2017.

The overall prevalence of uterine rupture among mothers who gave birth in Amhara Region referral hospitals was found to be 125 (16.68%) 95%CI: [14.0%,19.2%]). The pattern of prevalence of uterine rupture in 5 consecutive years (2013, 2014, 2015, 2016, 2017) at Amhara Region referral hospitals, was 18.70%, 18%, 16.70%, 15.30%, 14.70% respectively (Fig 1) and out of ruptured cases 113 (90.40%) were with complete uterine rupture as shown in Fig 2 below.

### Factors associated with uterine rupture

In bivariable logistic regression analysis shows that, the age of the respondents age, distance >10km, patient come with referral paper, lack of ANC, parity, laboring for ≥24hours, obstructed labor, precipitated labor, destructive delivery, partograph utilization, CPD, birth weight, morbidly adherent placenta, Mothers with GDM, history of myomectomy/uterine surgery, previous caesarean delivery, instrumental delivery, gestational, induction/ augmentation, any Obstetric procedure, those with traumatic vaginal birth, twins/multiple pregnancy were associations with uterine rupture at p-value less than 0.05.

As it is presented in the Table 3, distance from health facility > 10km ((AOR = 2.44, 95% CI (1.13, 5.28)), Parity between two and four (AOR = 7.26;95% (3.06,17.22)) and five and above (AOR = 12.55;95% CI (3.64,43.20)), duration of labor higher than 24 hours (AOR = 3.44, 95%CI (1.49, 7.92)), mothers who came with referral paper (AOR = 2.94, 95%CI (1.28, 6.55)) were significantly associated factors. Among care given/omitted by health care providers, induction/augmentation of labor (AOR = 2.34, 95%CI (1.15, 4.72)), lack of parthograph use (AOR = 5.21, (95%CI (2.72, 9.97)), Obstetric procedure (AOR = 2.54, 95%CI (1.09, 5.91)) and destructive delivery (AOR = 5.18, 95%CI (1.22, 20.08)) were significantly associated with uterine rupture in multivariable logistic regression model.

**Table 3:** Bivariable & Multivariable logistic regression result of factor associated with uterine rupture among women delivered in Amhara region referral hospitals from January 2013 to Dec 2017 (n = 750).

Variables	Uterine rupture		COR(95% CI)	AOR(95% CI)
	Yes	No		
Age				
≤18yrs	2	19	1	1
19-35yrs	98	549	1.69(1.01–2.84)*	1.01(0.12,8.28)
≥35yrs	25	57	4.17(2.60–6.67)*	1.46(0.69,3.10)
Distance				
≤10km	12	217	1	1
>10km	113	408	5.00(2.67,9.18)*	2.44(1.13,5.28)*
Parity				
Primipara	8	227	1	1
II-IV	102	374	7.73 (3.69,16.19)*	7.26(3.06.17.22)*
≥V	15	24	17.73(6.82,46.11)*	12.55(3.64, 43.20)*
ANC				
Yes	96	566	1	1
No	29	59	2.898(1.768,4.751)*	1.94(0.79.4.72)
Duration of labor				
<24hrs	84	577	1	1
≥24hrs	41	48	5.86(3.65,9.47)*	3.44(1.49,7.92)
Mother came with referral paper				
Yes	112	394	5.05(2.78,9.16)*	2.94(1.28,6.55)*
No	13	231	1	1
Gestational age				
<37 wks	12	92	1	1
37-42wks	100	515	1.48(1.07,2.81)*	1.32(0.62,2.80)
≥42wks	13	18	5.53(2.17,14.07)*	1.37(0.28,6.64)
Labor induced or augmented				
Yes	45	145	1.86(1.24,2.80)*	2.34(1.15,4.72)*
No	80	480	1	1
Obstructed labor				
Yes	59	33	16.03(9.82,26.48)*	4.88(2.22,10.70)*
No	66	592	1	1
Precipitated labor				
Yes	15	22	3.73(1.88,7.42)*	3.59(1.10,11.77)*
No	110	603	1	1
Instrumental delivery				
Yes	34	97	2.03(1.29,3.18)*	1.31(0.71,2.43)
No	91	528	1	1
Destructive delivery				
Yes	5	8	3.21(1.03,10.00)*	5.18(1.22,20.08)*
No	120	617	1	1
Traumatic vaginal birth				
Yes	39	128	1.76(1.15,2.69)*	1.06(0.456,2.48)
No	86	497	1	1
Parthograph used				
Yes	43	499	1	1
No	82	126	7.552(4.97,11.47)*	5.21(2.72,9.97)*
Obstetric procedure				
Yes	23	31	2.61(2.49,7.99)*	2.54(1.09,5.91)*
No	112	394	1	1
CPD				
Yes	68	63	10.64(6.87,16.47)*	4.08(1.99,8.33)*
No	57	562	1	1
Birth weight the baby				
<4000gm	110	602	1	1
> = 4000gm	15	23	3.56(1.73,6.71)*	1.83(0.76,4.43)
Morbidly adhere Placenta				
Yes	6	9	3.45(1.20,9.78)*	9.00(2.46,27.11)*
No	119	616	1	1
Gestational diabetes				
Yes	16	19	4.68(2.33,9.37)*	5.78(1.12,20.00)*
No	109	606	1	1
Twins/multiple pregnancy				
Yes	15	23	3.57(1.81,7.06)*	1.86(0.42,8.34)
No	110	602	1	1
Previous uterine surgery				
Yes	6	10	3.10(1.11,8.71)*	5.00(1.33,18.73)*
No	119	615	1	1
Previous caesarean delivery				
Yes	20	36	3.11(1.74,5.59)*	4.90 (2.13,11.26)*
No	105	589	1	1

NB: 1 = Reference

*p-value<0.05

In other hand, obstructed labor (AOR = 4.88, 95%CI (2.22,10.70)), precipitated labor (AOR = 3.59, 95%CI (1.10,11.77)), CPD (AOR = 4.08, 95%CI (1.99,8.33)), Morbidly adherent Placenta (AOR = 9.00, 95%CI (2.46, 27.11)) and gestational DM (AOR = 5.78(1.12,20.00)) were among characteristics and complications during pregnancy and labor which found to be significantly predict the prevalence of uterine rupture. From factors related with previous obstetrics history, previous myomectomy/other uterine surgery (AOR = 5.00, 95%CI (1.33, 18.73)) and last caesarian delivery (AOR = 4.90, 95% CI (2.13, 11.26)) were significantly associated with uterine rupture in multivariable logistic regression model at p-value less than 0.05 (Table 3).

## Discussion

A total of 125 (16.68%, 95% (14.0%, 19.2%) women experienced uterine rupture which is in line with Burkina Faso (18%)[11]. But, by far higher when compared with that from a study done at Debremarkos (9.5%)[1], Debremarkos 2017 2.24%[12], rural Ethiopia (3.7%)[13], Mizan Aman General Hospital (1.6%)[14], Somalia (0.7%)[15], Mali[7], Benin Nigeria (0.58%)[16] and Israel (6.7%)[17]. Differences in delivery services coverage, accessibility of the facilities as well as the availability of skilled personnel and medical supplies as the study setting differs could contribute to variation observed.

The odds of getting uterine rupture in mothers who were para two to four were seven (AOR = 7.26;95% (3.06,17.22)), and five and above were twelve (AOR = 12.55;95% CI (3.64,43.20) times higher as compared to primipara. This finding is supported by other studies done in western Uganda [18] and Suhul General Hospital, Shire [19] which could be because the increment in parity ends up with the weakening of the uterine muscle, which could not be resistant to prolonged and powerful labor, especially in grand multipara women. As a result, the uterine rupture is more likely than primiparous women than parous women.

Those mothers who were living 10 and above kilometers away from health institution they received care were two times (AOR = 2.44, 95%CI (1.13, 5.28)) more likely exposed to uterine rupture than their counterparts as also reported in the studies done at Debre Markos Hospital [1], western Uganda [18] and rural Uganda [20]. The possible explanation might be, as the distance from health facility increases, the women suffer for a long time to reach health facility while they are in labor. Therefore, this will increase the duration of labor as a whole and increase the probability of acquiring uterine rupture. The weak network of the road has a prominent impact on the effectiveness of an intervention.

Those mothers who were laboring for twenty-four hours and above had three times(AOR = 3.44;95%CI:1.49,7.92) higher likelihood of having uterine rupture as compared to those staying in labor less than 24 hours. This finding supports the evidence of other studies conducted at Suhul General Hospital Shire[18], Mizan Aman General hospital[14]. From this, one can understand that the threat of prolonged labor in Amhara region remains high and ending up with uterine rupture. Enormous challenges of accessing quality services arising from three delays including, delays seeking care, reaching facilities and getting care could have influenced it.

Whereas, mothers who referred from other health institution had three times (AOR = 2.94; 95% CI: 1.28, 6.55) increased probability of having uterine rupture than those who came for care directly by themselves. The study done in Debre Markos supported this finding[1]. The clients who come to get services from tertiary health facilities are in a complicated condition as compared to those come from their home directly to these facilities. Therefore, these complications enhance the likely hood of developing uterine rupture. Those with labor abnormality and failed to be referred early due to factors related to road distance or health care provider pertaining with early referral end up with rupture. But this finding contradicts with the study conducted at Mizan Aman General hospital as those came without referral were in increased risk of uterine rupture[14]. The difference possibly explained by good referral system, accessibility of roads, and skill of health care providers to identify labor abnormalities, awareness of mothers towards institutional delivery, demographic and time variation.

Obstructer labor had a significant association with uterine rupture. Mothers diagnosed with obstructed labor were five times (AOR = 4.88; 95%CI: 2.22, 10.70) more likely exposed to uterine rupture than mothers without obstructed labor. This finding was also witnessed in other studies conducted in Debre Markos Referral Hospital [1], Suhul General Hospital Shire [19], and rural Uganda [20]. Obstructed labor is one of the top underlying cause of the uterine rupture in Amhara region Ethiopia. Presence of health facilities with emergency services in long distance from primary or home of the clients and lack of services compressive emergency obstetric care in some facilities might have enhanced the problem. Low utilization of the available maternal health services like ANC, even if it is free service at the national level also played its role by minimizing health care need of the individuals.

Likewise, mothers who had precipitated labor had four times (AOR = 3.59; 95%CI: 1.10, 11.77) increased odds of having uterine rupture than their counterparts. Whenever labor precipitated, the risk of genital tract trauma and extension to the uterus is highly likely since it is sudden to support, poor counseling about risk of subsequent precipitated labor, poor labor follow up practice. Those who had destructive operative delivery had five odds of having uterine rupture than without destructive deliveries (AOR = 5.18;95%:1.22,20.08). This might be explained due to the iatrogenic injury of the genital tract and the uterus which is highly susceptible to rupture in prolonged and obstructed labor, the poor skill of the operator.

Failing to follow lobar by partograph in the course of labor had five times (AOR = 5.21, 95%CI: 2.72, 9.97) increased odds of uterine rupture as compared to those who followed by partograph as also reported in other studies[1,17]. Partograph utilization to monitor the progress of the feto-maternal condition will enable to prevent the occurrence of prolonged labor. As a result, it will also prevent uterine rupture. However, failure to use partograph could occur due to various factors as we can see in this report. Negligence of health professionals to practice it or poor understanding of the benefit of partograph or either low human power may contribute to underutilization of partograph, which in return increased the magnitude of uterine rupture.

Mothers diagnosed with Cephalo Pelvic Disproportion had four times (AOR = 4.08; 95%CI: 1.99, 8.33) more likely to have the uterine rupture. The problem of pelvis, fetus or both leads to CPD, repeated contraction and relaxation without advancement of labor exhaustion and end up with uterine rupture, lack of skilled attendant to peak CPD early, poor childhood nutritional status.

Morbidly adhere Placenta had nine times (AOR = 9.00; 95%:2.46, 27.11) increased risk of uterine rupture than their counterparts. The finding supports the evidence reported from UK[20]. If there is placenta previa, the risk of the adherent placenta increases, which in return enhance labor complications. However, this finding opposes the study carried out at Columbia University[21]. Early diagnosis of those cases with Doppler sonography, close follow up and elective management before she went to the labor and delivery process, healthcare setup, quality of care providers could have minimized the risk of uterine rupture in the latter study.

Gestational diabetic’s militias had six times (AOR = 5.78; 95%CI: 1.12, 20.00) odds of having uterine rupture than non-diabetics. The risk of feto-pelvic disproportion and obstructed labor is high due to fat accumulation on fetal shoulder in diabetic patient. That leads to shoulder dystocia; difficult prolonged labor and then finally it ends up with uterine rupture. Previous myomectomy or other uterine surgeries had five times (AOR = 5.00; 95%CI: 1.33, 18.73) increased the likelihood of uterine rupture than without surgery. It is supported by the findings that an increased number of prior uterine surgery lead to an increase in the probability of uterine rupture. But this finding contradicts the study conducted in Columbia University[21], which could be may because of difference in health care system.

Among mothers who were induced or augmented odds of getting uterine rupture is two times (AOR = 2.34, 95%:1.15, 4.72) more likely than their counterparts which are in line with other studies from Norway [22] and UK [23]. Uterotonics drugs increases hyper stimulation of the uterus leads to rupture, if not used appropriately otherwise. Widely use of misoprostol for mothers with post-term pregnancy, term premature rupture of membrane, pre-eclampsia or eclampsia, and other cases are implemented, which predict the problem of having a uterine rupture. Those Mothers attended with any obstetric procedure had three times (AOR = 2.54; 95%:1.09, 5.91) more likely to have uterine rupture as compared to without procedure. This is supported by the study done at Shire[19]. Wherever there is uterine manipulation, and this happens by poorly skilled health care providers and unforeseen adherent placenta, the risk of uterine rupture increases.

Among mothers who had previous cesarean deliveries the odds of being rupture was five times (AOR = 4.90, 95%:2.13, 11.26) more likely than their counterparts which was similar with the study conducted in Nigeria[24] and UK[23]. The risk of scar dehiscence and silent dehiscence increases in cases of previous scars, which made them stay at higher risk of uterine rupture. If the patient request for vaginal birth after caesarian section within short inter-pregnancy intervals which might occur if poor counseling is given, the risk encountering uterine rupture will be significant.

## Conclusions

This study indicates that the prevalence of uterine rupture was found to be high. A more careful strategy to counter obstructed labor, consistent use of partograph, along with a high index of anticipation of imminent uterine rupture and quick referral to a well-equipped center is recommended.

## Acronyms

ANC: Antenatal Care
AOR: Adjusted Odd Ratio
CI: Confidence Interval
COR: Cruds Odd Ratio
CPD: Cephalo Pelvic Disproportion
GDM: Gestational Diabetes Mellitus
IRB: Institutional Review Board
MM: Maternal Mortality
SD: Standard Deviation
SSA: Sub-Saharan African
SVD: Spontaneous Vertex Delivery
